# Optimal infused CD34^+^ cell dose in multiple myeloma patients undergoing upfront autologous hematopoietic stem cell transplantation

**DOI:** 10.1038/s41408-024-01165-w

**Published:** 2024-10-31

**Authors:** Oren Pasvolsky, Curtis Marcoux, Denái R. Milton, Babar Pal, Mark R. Tanner, Qaiser Bashir, Samer Srour, Jaehyun Lee, Neeraj Saini, Paul Lin, Jeremy Ramdial, Yago Nieto, Guilin Tang, Yosra Aljawai, Partow Kebriaei, Melody R. Becnel, Hans C. Lee, Krina K. Patel, Sheeba K. Thomas, Robert Z. Orlowski, Elizabeth J. Shpall, Richard E. Champlin, Muzaffar H. Qazilbash

**Affiliations:** 1 Department of Lymphoma and Myeloma, The University of Texas MD Anderson Cancer Center, Houston, TX USA; 2 Division of Hematology, Dalhousie University, Halifax, NS Canada; 3 Department of Biostatistics, The University of Texas MD Anderson Cancer Center, Houston, TX USA; 4 Department of Stem Cell Transplantation and Cellular Therapy, The University of Texas MD Anderson Cancer Center, Houston, TX USA; 5 Department of Hematopoietic Biology and Malignancy, University of Texas MD Anderson Cancer Center, Houston, TX USA; 6 Department of Hematopathology, The University of Texas MD Anderson Cancer Center, Houston, TX USA

**Keywords:** Myeloma, Stem-cell therapies

## Abstract

Autologous transplantation remains the standard of care for eligible multiple myeloma (MM) patients, yet optimal CD34^+^ cell dose remains unclear. We conducted a retrospective study on MM patients undergoing upfront transplant between 2005 and 2021 and divided them into low (≤2.5 × 10^6^ cells/kg) and high (>2.5 × 10^6^ cells/kg) CD34^+^ dose groups. We included 2479 patients, 95 in the low CD34^+^ group and 2384 in the high CD34^+^ group. Patients in the low CD34^+^ group were older (63.2 vs 61.1 years, *p* = 0.013), more often had R-ISS III (19% vs 9%, *p* = 0.014), received plerixafor (60% vs 35%, *p* < 0.001) and transplanted after 2009 (88% vs 80%, *p* = 0.047). Time to neutrophil and platelet recovery was longer in the low CD34^+^ group. Median PFS and OS were lower in the low CD34^+^ group (31.6 vs. 43.6 months, *p* = 0.011 and 76.4 vs. 108.2 months, *p* < 0.001, respectively). Evaluation of incrementally higher CD34^+^ dose did not show significant improvement in survival at thresholds >2.5 × 10^6^ cells/kg. Multivariable analysis affirmed that CD34^+^ >2.5 × 10^6^ cells/kg was associated with better PFS (HR 0.71, *p* = 0.008) and OS (0.59, *p* < 0.001). After propensity score matching, a CD34^+^ dose >2.5 × 10^6^ cells/kg remained a predictor of better OS (0.42, *p* < 0.001). In conclusion, CD34^+^ dose >2.5 × 10^6^ cells/kg was associated with improved survival, without any additional benefit at incrementally higher doses.

## Introduction

In the dynamic landscape of treatment for multiple myeloma (MM), autologous hematopoietic stem cell transplantation (auto-HCT) remains the standard of care for eligible newly diagnosed patients. Despite its decades-long use, the optimal CD34^+^ cell dose to infuse during auto-HCT is still not known.

The International Myeloma Working Group (IMWG) published recommendations in 2009 for the minimum and ideal collection and infusion thresholds for patients with MM undergoing auto-HCT. They recommended a minimum dose of ≥2 × 10^6^ CD34^+^ cells/kg and an optimal dose of 4–6 × 10^6^ CD34^+^ cells/kg for a single transplant [1]. These recommendations were based on heterogenous data that also included non-myeloma patients, and predated modern therapies. There is considerable variability in the impact of CD34^+^ cell dose on neutrophil and platelet engraftment in MM patients in the published literature. One study showed that a higher CD34^+^ cell dose was associated with a faster platelet recovery without a beneficial effect on neutrophil recovery [2]. In contrast, another study showed more rapid hematologic engraftment with higher CD34^+^ cell doses [3]. Interestingly, a third study showed no positive impact of a higher CD34^+^ cell dose on hematologic engraftment [4].

Similarly, there are contradictory reports on the impact of CD34^+^ cell dose on survival in MM. This is in contrast with studies in lymphoma where there is a direct correlation between a higher CD34^+^ dose and improved survival in patients undergoing auto-HCT [5–8]. One study demonstrated improved hematologic recovery and median overall survival (OS) in MM patients receiving ≥5 × 10^6^ CD34^+^ cells/kg [9], while a post-hoc analysis of the GOA trial demonstrated no discernable difference in progression-free survival (PFS) or OS across three CD34^+^ dose groups (<1.0 × 10^6^ CD34^+^ cells/kg, 1–1.9 × 10^6^ CD34^+^ cells/kg, and ≥2 × 10^6^ CD34^+^ cells/kg) [10]. Furthermore, a recent study that included 621 patients showed shorter median PFS and OS in patients that had ≥8 × 10^6^ CD34^+^ cell/kg collected for auto-HCT [11].

While there is variability in collection and infusion targets between transplant centers, clinical practice has remained largely unchanged for many years. With new therapeutic modalities for MM gaining favor, including increased use of quadruplet induction and earlier and more frequent use of CAR-T cells that can cause prolonged pancytopenia, the subject of optimal CD34^+^ cell dose in auto-HCT for MM has gained renewed interest [12, 13].

Given the lack of consensus on an optimal CD34^+^ cell dose, we sought to study the association of CD34^+^ cell dose on the outcomes of MM patients who underwent upfront auto-HCT.

## Materials and methods

### Study design and participants

We conducted a retrospective, single-center study on newly diagnosed patients with MM who underwent upfront auto-HCT between 2005 and 2021. Data were obtained from our institution’s transplantation database and chart-based review. We included patients with available information on the infused CD34^+^ cell dose at auto-HCT. Primary outcomes included neutrophil and platelet engraftment, PFS, and OS. Secondary outcomes included response rates and the depth of post-transplant response. The study was conducted after approval by our Institutional Review Board and in accordance with the Declaration of Helsinki and the 1996 Health Insurance Portability and Accountability Act.

We evaluated response rates according to the criteria outlined by the IMWG [14]. Minimal residual disease (MRD) status in bone marrow samples was determined utilizing an eight-color next-generation flow cytometry (NGF) technique with a sensitivity of 1/10^−5^ cells (0.001%) through the acquisition and analysis of at least of two million events. Fluorescence in situ hybridization (FISH) analysis was conducted to detect high-risk cytogenetic abnormalities, specifically *t*(4;14), *t*(14;16), del(17p), and 1q21 gain or amplification, utilizing IGH::FGFR3 dual-color dual-fusion probes, IGH::MAF dual-color dual-fusion probes, TP53/CEP17 dual-color probes, and CDKN2C/CKS1B dual-color probes.

### Statistical methods

Neutrophil and platelet engraftment as well as other continuous measures were summarized by medians and ranges and evaluated by the Wilcoxon rank-sum test. Categorical variables were summarized using frequencies and percentages and assessed by Fisher’s exact test or its generalizations. OS time was computed from the date of auto-HCT to the last known vital sign, with censoring for patients alive at the last follow-up. PFS time was computed from the date of auto-HCT to the date of disease progression, death (if without disease progression), or the last follow-up. Patients alive with no disease progression were censored at their last follow-up date. OS and PFS were estimated using the Kaplan–Meier method, with group differences assessed by the log-rank test. Associations between OS/PFS and measures of interest were assessed using Cox proportional hazards regression models.

The web application *Cutoff Finder* [15] was used to find the optimal cutoff point for CD34^+^ cell dose infusion with survival based on the log-rank test. Patients were then categorized into low (≤2.5 × 10^6^ cells/kg) and high (>2.5 × 10^6^ cells/kg) CD34^+^ groups based infused CD34^+^ cell quantity. There was considerable disparity between patient number in the low (*N* = 95) and high (*N* = 2384) CD34^+^ groups. To address the difference in sample sizes, 2:1 nearest-neighbor matching was employed [16, 17]. This matching method, utilizing propensity scores derived from a logistic regression model, ensured comparability for subsequent analysis. For each patient in the “low” dose group, control matches from the “high” dose group were selected one at a time based on a descending order of the distance measure. Matching variables included age at auto-HCT, year of auto-HCT (<2010, ≥2010), cytogenetic risk (standard, high, unknown), Revised International Staging System (R-ISS) stage (I, II, III, unknown), Hematopoietic Cell Transplantation (HCT)-specific Comorbidity Index (HCT-CI) (≤3, >3), induction treatment [bortezomib, lenalidomide, and dexamethasone (VRD), bortezomib, cyclophosphamide, and dexamethasone (VCD), bortezomib and dexamethasone (VD), carfilzomib, lenalidomide, and dexamethasone (KRD), immunomodulator and dexamethasone (ImiD + Dexa), bortezomib thalidomide and dexamethasone (VTD), or other], mobilization with/without chemotherapy (yes, no, unknown), conditioning regimen (melphalan, busulfan and melphalan, other), and maintenance treatment use (yes, no). Of note, we did not use plerixafor as a matching variable since it generally has not been shown to impact survival outcomes [18].

Nearest-neighbor matching was performed using the MatchIt package in R (MatchIt: Nonparametric Preprocessing for Parametric Causal Inference). All other statistical analyses were conducted using SAS 9.4 for Windows (SAS Institute Inc., Cary, NC) with a significance level of 5%. No adjustments for multiple testing were made.

## Results

### Patient, disease, and treatment characteristics

A total of 2479 patients were included in our analysis, 95 in the low CD34^+^ dose group and 2384 in the high CD34^+^ dose group. There were 21 patients who received <2 × 10^6^ cells/kg in the low CD34^+^ dose group. Median age at auto-HCT in the entire cohort was 61 years (range 25–83), and 59% were males (*n* = 1460). In the low CD34^+^ group, patients were older on average (63 vs 61 years, *p* = 0.013), a higher percentage had R-ISS III (19% vs 9%, *p* = 0.014), more often received plerixafor for mobilization (60% vs 35%, *i* < 0.001) and more often transplanted after 2009 (88% vs 80%, *p* = 0.047). In both CD34^+^ dose groups, VRD (28% and 31%, respectively) and KRD (18% and 15%, respectively) were the most commonly used induction regimens. After matching, only R-ISS (*p* = 0.048) remained significantly different between the low and high CD34^+^ dose groups. Patient characteristics are summarized in Table 1. Patient characteristics for the matched cohorts are presented in Supplementary Table 1.

**Table 1:** Patient characteristics – All patients and by CD34^+^ dose group.

		CD34^+^ dose group		
Measure	All	≤2.5 × 10^6^ cells/kg	>2.5 × 10^6^ cells/kg	*p*-value
	(*N* = 2479)	(*N* = 95)	(*N* = 2384)	
**Gender,** ***n*** **(%)**				0.24
Female	1019 (41)	45 (47)	974 (41)	
Male	1460 (59)	50 (53)	1410 (59)	
**Race,** ***n*** **(%)**				0.13
Black	445 (18)	11 (12)	434 (19)	
Non-Black	1989 (82)	82 (88)	1907 (81)	
Unknown	45	2	43	
**Age at auto-HCT (years)**				0.013
Median (range)	61 (25–83)	63 (32–78)	61 (25–83)	
**Year of auto-HCT,** ***n*** **(%)**				0.047
<2010	487 (20)	11 (12)	476 (20)	
≥2010	1992 (80)	84 (88)	1908 (80)	
**R-ISS,** ***n*** **(%)**				0.002
I	567 (35)	12 (19)	555 (36)	
II	895 (56)	40 (63)	855 (55)	
III	150 (9)	12 (19)	138 (9)	
Unknown	867	31	836	
**Light chain type,** ***n*** **(%)**				0.33
Kappa	1620 (66)	59 (63)	1561 (66)	
Lambda	834 (34)	34 (36)	800 (34)	
Biclonal	11 (<1)	1 (1)	10 (<1)	
Unknown	14	1	13	
**Cytogenetic risk,** ***n*** **(%)**				0.32
Standard	1704 (74)	59 (69)	1645 (74)	
High	597 (26)	26 (31)	571 (26)	
Unknown	178	10	168	
**LDH,** ***n*** **(%)**				0.72
Normal	1360 (83)	49 (82)	1311 (83)	
>ULN	272 (17)	11 (18)	261 (17)	
Unknown	847	35	812	
**Creatinine**				0.07
≤2	1964 (85)	70 (78)	1894 (85)	
>2	359 (15)	20 (22)	339 (15)	
Unknown	156	5	151	
**HCT-Cl,** ***n*** **%**				0.47
≤3	1875 (76)	69 (73)	1806 (76)	
>3	601 (24)	26 (27)	575 (24)	
Unknown	3	0	3	
**Chemotherapy-mobilization**				0.52
No	2030 (87)	83 (90)	1947 (87)	
Yes	293 (13)	9 (10)	284 (13)	
Unknown	156	3	153	
**Plerixafor use**				<0.001
No	1475 (64)	36 (40)	1439 (65)	
Yes	843 (36)	55 (60)	788 (35)	
Unknown	161	4	157	
**Infused CD34**^**+**^ **count**				<0.001
Median (range)	4.1 (0.6–26.6)	2.3 (0.6–2.5)	4.2 (2.5–26.6)	
Mean	4.5	2.2	4.6	
**Induction regimens,** ***n*** **%**				
KRD	364 (15)	17 (18)	347 (15)	0.37
ImiD+Dexa	340 (14)	10 (11)	330 (14)	0.45
VTD	104 (4)	5 (5)	99 (4)	0.60
VCD	301 (12)	11 (12)	290 (12)	1.00
VD	335 (14)	10 (11)	325 (14)	0.45
VRD	763 (31)	27 (28)	736 (31)	0.65
Missing/Other	2	0	2	
**Conditioning regimen,** ***n*** **(%)**				0.014
Mel	2023 (82)	84 (88)	1939 (81)	
Bu/Mel based	309 (12)	11 (12)	298 (13)	
Other	147 (6)	0	147 (6)	
**Response prior to auto-HCT,** ***n*** **(%)**				
CR/sCR	329 (13)	16 (17)	313 (13)	0.28
VGPR	1065 (43)	40 (42)	1025 (43)	0.92
PR	1022 (41)	38 (40)	984 (41)	0.83
SD	62 (3)	1 (1)	61 (3)	0.73
PD	1 (<1)	0	1 (<1)	-
**MRD status prior to auto-HCT,** ***n*** **(%)**				0.32
Negative	669 (42)	33 (48)	636 (42)	
Positive	925 (58)	36 (52)	889 (58)	
Not done/unknown	885	26	859	
**Any maintenance,** ***n*** **(%)**				0.25
Yes	1762 (71)	73 (77)	1689 (71)	
No	717 (29)	22 (23)	695 (29)	
**Maintenance therapy,** ***n*** **(%)**				0.20
Rev ± Dexa	1347 (77)	51 (70)	1296 (77)	
Non-Rev^a^	314 (18)	16 (22)	298 (18)	
Rev/Elo	87 (5)	6 (8)	81 (5)	

*auto-HCT* autologous hematopoietic stem cell transplant, *Bu/Mel* busulfan, melphalan, *CR* complete response, *Dexa* dexamethasone, *Elo* elotuzumab, *HCT-CI* hematopoietic cell transplantation-specific comorbidity index, *ImiD* immunomodulatory drug, *KRD* carfilzomib, lenalidomide, dexamethasone, *LDH* lactate dehydrogenase, *Mel* melphalan, *MRD* minimal residual disease, *n* number, *PR* partial response, *R-ISS* Revised International Staging Systems, *Rev* lenalidomide, *sCR* stringent complete response, *SD* stable disease, *ULN* upper limit of normal, *VCD* bortezomib, cyclophosphamide, dexamethasone, *VD* bortezomib, dexamethasone, *VGPR* very good partial response, *VRD* bortezomib, lenalidomide, dexamethasone, *VTD* bortezomib, thalidomide, dexamethasone.

^a^Includes: Dara alone or in combination (*n* = 19); Thalidomide or pomalidomide, with or without Dexa (*n* = 64); thalidomide or pomalidomide +PI (88); interferon-based (*n* = 1); PI with or without Dexa (*n* = 142).

### Engraftment

In the low CD34^+^ dose group, neutrophil recovery (absolute neutrophil count (ANC) ≥ 500 × 10^8^ cells/L) occurred at a median of 12 days after auto-HCT, compared to 11 days in the high CD34^+^ dose group (*p* < 0.001). Platelet recovery to ≥20 × 10^9^ cells/L and ≥50 × 10^9^ occurred at a median of 14 and 18 days in the low CD34^+^ dose group, in contrast to 11 and 14 days in the high CD34^+^ dose group (*p* < 0.001). Only one patient in the entire cohort, in the high CD34^+^ dose group, had engraftment failure. Individuals in the low CD34^+^ dose group received more red blood cell (RBC) transfusions, with a median of two units compared to one unit in the high CD34^+^ dose group (*p* < 0.001). Similarly, those in the low CD34^+^ dose group received more platelet transfusions, with a median of three units compared to two units in the high-dose CD34^+^ dose group (*p* < 0.001). Engraftment outcomes are summarized in Supplementary Table 2.

Patients in the high CD34^+^ group had a shorter median duration of hospitalization for auto-HCT compared to the low CD34+ group [16 (range 0–94) days and 17 (range 0–38) days, respectively; *p* = 0.007]. This translates to an estimated saving of 2700 US dollars per patient (i.e., average daily cost of hospital stay) in the high CD34^+^ group.

### Response and MRD assessment

**Fig. 1:**
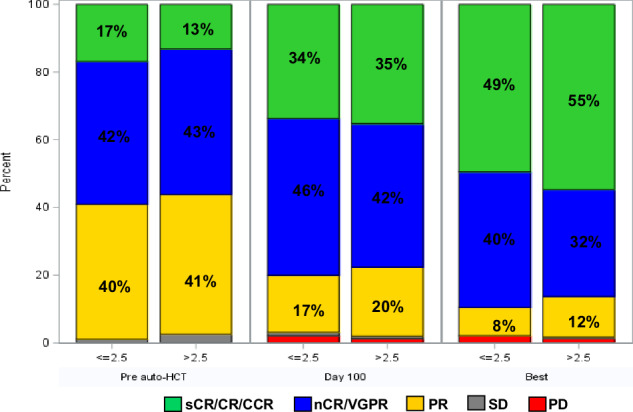
Hematologic response rates pre-auto-HCT, at day 100 after auto-HCT, and at the best post-auto-HCT response in MM patients who underwent auto-HCT with either ≤2.5 × 10^6^ CD34^+^ cells/kg or >2.5 × 10^6^ CD34^+^ cells/kg.

There were no significant differences in day-100 and best post-auto-HCT responses based on CD34^+^ cell dose (Fig. 1). In the low CD34^+^ dose group, day-100 ≥complete response (CR) and ≥very good partial response (VGPR) response rates were 34% and 80%, respectively, and at best post-transplant response ≥CR and ≥VGPR response rates improved to 49% and 89%, respectively. In the high CD34^+^ group, day-100 ≥ CR and ≥VGPR response rates were 35% and 78%, respectively, and at best post-transplant response ≥CR and ≥VGPR response rates improved to 55% and 86%, respectively. MRD negative ≥VGPR was seen in 72% and 65% of evaluable patients in the low and high CD34^+^ dose groups, respectively. Similarly, there was no significant difference in post-transplant response or MRD rates in the propensity-matched patients (Supplementary Table 3).

### Survival outcomes

**Fig. 2:**
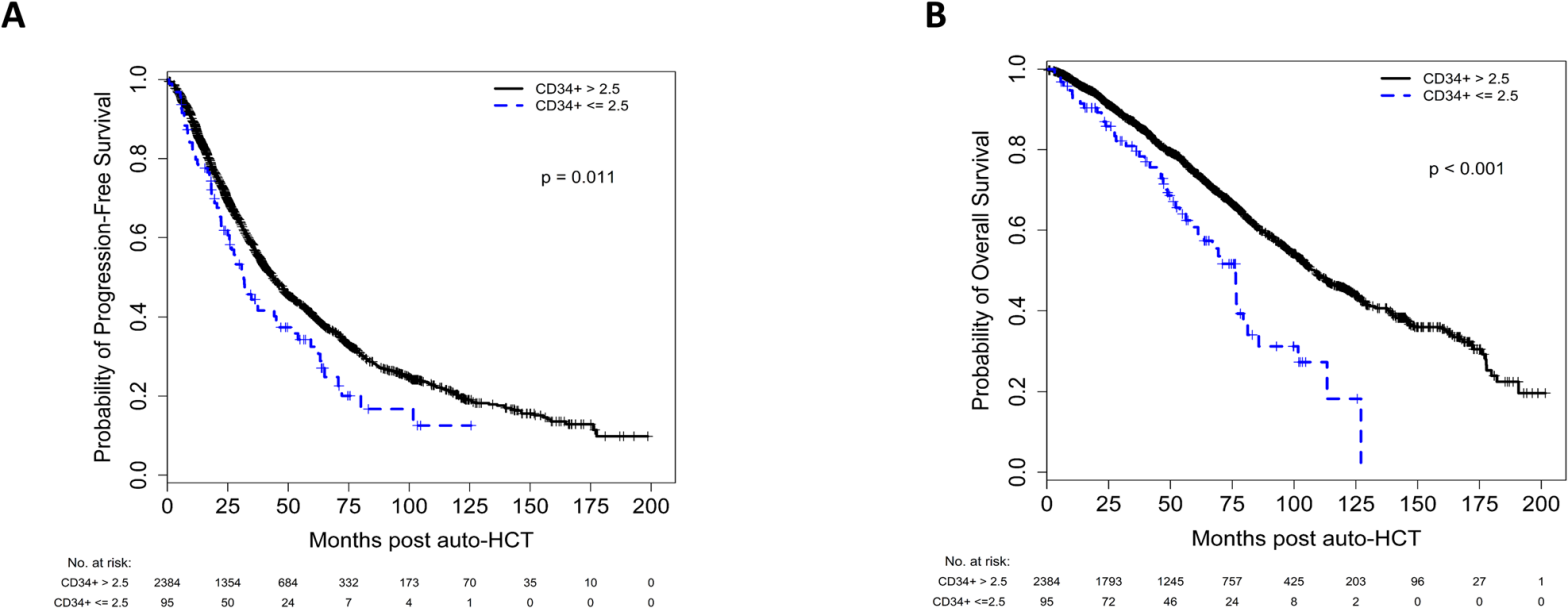
Patient outcomes. Progression-free survival (**A**) and overall survival (**B**) in MM patients who underwent auto-HCT with either ≤2.5 × 10^6^ CD34^+^ cells/kg or >2.5 × 10^6^ CD34^+^ cells/kg.

Median follow-up for the entire cohort was 51.9 months (range 0.2–201.6). Median PFS was 31.6 and 43.6 months (*p* = 0.011) in the low and high-dose CD34^+^ dose groups, respectively (Fig. 2A). The median OS was 76.4 and 108.2 months (*p* < 0.001) months in the low and high CD34^+^ dose groups, respectively (Fig. 2B). Five-year OS rate was 61% and 74% for low and high CD34^+^ dose groups, respectively. We also assessed the impact of incremental CD34^+^ cell dose on PFS and OS, up to 6 × 10^6^ cells/kg. As shown in Supplementary Table 4, in univariate analysis there was no significant correlation between doses above 2.5 × 10^6^ cells/kg and either PFS or OS. Day-100 non-relapse mortality (NRM) was 0% and 1% (*p* = 0.47) in the low and high CD34^+^ dose groups, respectively.

In multivariable analysis for PFS, CD34^+^ cell dose >2.5 × 106 cells/kg (hazard ratio [95% CI] 0.71 [0.55–0.91], *p* = 0.008) was associated with superior PFS. Other variables associated with superior PFS included auto-HCT after 2009 (0.79 [0.68–0.91], *p* = 0.002), use of KRD as the induction regimen (0.76 [0.61–0.94], *p* = 0.013) and achieving MRD negative ≥VGPR prior to auto-HCT (0.59 [0.50–0.69, *p* < 0.001)]. In contrast, R-ISS stage III (1.62 [1.25–2.11], *p* < 0.001) compared to R-ISS stage I, lambda light chain type (1.24 [1.11–1.39], *p* < 0.001), high-risk cytogenetics (1.93 [1.67–2.23], *p* < 0.001) and the use of chemotherapy for mobilization (1.24 [1.07–1.45], *p* = 0.005) were associated with inferior PFS (Table 2). Univariate analysis for PFS is shown in Supplementary Table 5.

**Table 2:** Summary of progression-free survival – multivariable assessments.

Measure	Hazard ratio (95% CI)	*p*-value
**CD34**^**+**^ **(×10**^**6**^ **cells/kg)**		
>2.5 vs. ≤2.5	0.71 (0.55, 0.91)	0.008
**Age at auto-HCT**		
Continuous	1.01 (1.01, 1.02)	<0.001
**Year of auto-HCT**		
≥2010 vs. <2010	0.79 (0.68, 0.91)	0.002
**R-ISS**		
II vs. I	1.15 (0.98, 1.34)	0.08
III vs. I	1.62 (1.25, 2.11)	<0.001
Unknown vs. I	1.48 (1.25, 1.75)	<0.001
**Light chain type**		
Lambda vs. Kappa	1.24 (1.11, 1.39)	<0.001
Biclonal vs. Kappa	0.69 (0.33, 1.45)	0.32
Unknown vs. Kappa	1.37 (0.51, 3.70)	0.53
**Cytogenetic risk**		
High vs. Standard	1.93 (1.67, 2.23)	<0.001
Unknown vs. Standard	1.20 (0.98, 1.47)	0.07
**LDH**		
> ULN vs. Normal	1.12 (0.92, 1.35)	0.26
Unknown vs. Normal	0.97 (0.85, 1.10)	0.59
**Chemotherapy-mobilization**		
Yes vs. No	1.24 (1.07, 1.45)	0.005
Unknown vs. No	0.62 (0.25, 1.57)	0.31
**Induction treatment**		
KRD vs. Other	0.76 (0.61, 0.94)	0.013
**Plerixafor use**		
Yes vs. No	0.93 (0.81, 1.07)	0.30
Unknown vs. No	1.68 (0.68, 4.17)	0.26
**MRD negative** ≥ **VGPR prior to auto-HCT**		
Yes vs. No	0.59 (0.50, 0.69)	<0.001
**MRD negative** ≥ **VGPR at best post-transplant response**^**a**^		
Yes vs. No	0.82 (0.68, 0.99)	0.040
**Maintenance therapy**^**a**^		
Yes vs. No	0.87 (0.77, 0.99)	0.037

*auto-HCT* autologous hematopoietic stem cell transplant, *CI* confidence interval, *KRD* carfilzomib, lenalidomide, dexamethasone, *LDH* lactate dehydrogenase, *MRD* minimal residual disease, *R-ISS* Revised International Staging Systems, *ULN* upper limit of normal, *VGPR* very good partial response.

^a^Included in the model as a time-dependent covariate.

In multivariable analysis for OS, CD34^+^ cell dose >2.5 × 106 cells/kg (0.59 [0.44–0.79], *p* < 0.001) was associated with superior OS. Other variables associated with superior OS were achieving a CR at the best response (0.50 [0.43–0.59], *p* < 0.001), and the use of maintenance therapy (0.69 [0.59–0.81], *p* < 0.001). In contrast, R-ISS stage III (2.08 [1.46–2.97, *p* < 0.001)] compared to R-ISS stage I, lambda light chain type (1.27 [1.10–1.47], *p* < 0.001), high-risk cytogenetics (2.15 [1.78–2.60], *p* < 0.001) and HCT-CI > 3 (1.41 [1.20–1.65], *p* < 0.001) were associated with worse OS (Table 3). After propensity score matching, a CD34^+^ dose of >2.5 × 106 cells/kg remained associated with better OS (0.42 [0.28–0.63, *p* < 0.001)] (Table 4). Univariate analysis for OS is shown in Supplementary Table 6.

**Table 3:** Summary of overall survival – multivariable assessments.

Measure	Overall survival	
	Hazard ratio (95% CI)	*p*-value
**CD34**^**+**^ **(×10**^**6**^ **cells/kg)**		
>2.5 vs. ≤2.5	0.59 (0.44, 0.79)	<0.001
**Age at auto-HCT**		
Continuous	1.03 (1.02, 1.03)	<0.001
**Year of auto-HCT**		
≥2010 vs. <2010	1.00 (0.83, 1.20)	0.99
**R-ISS**		
II vs. I	1.27 (1.00, 1.60)	0.046
III vs. I	2.08 (1.46, 2.97)	<0.001
Unknown vs. I	2.16 (1.71, 2.73)	<0.001
**Light chain type**		
Lambda vs. Kappa	1.27 (1.10, 1.47)	<0.001
Biclonal vs. Kappa	0.91 (0.37, 2.19)	0.83
Unknown vs. Kappa	1.84 (0.59, 5.77)	0.30
**Cytogenetic risk**		
High vs. Standard	2.15 (1.78, 2.60)	<0.001
Unknown vs. Standard	1.33 (1.04, 1.69)	0.024
**LDH**		
>ULN vs. Normal	1.25 (0.98, 1.60)	0.07
Unknown vs. Normal	0.97 (0.81, 1.14)	0.68
**Creatinine**		
>2 vs. ≤2	1.14 (0.93, 1.38)	0.20
Unknown vs. ≤2	0.80 (0.60, 1.07)	0.14
**HCT-CI**		
>3 vs. ≤3	1.41 (1.20, 1.65)	<0.001
Unknown vs. ≤3	2.14 (0.30, 15.32)	0.45
**Induction treatment**		
KRD vs. Other	0.79 (0.55, 1.12)	0.19
**MRD negative** ≥ **VGPR prior to auto-HCT**		
Yes vs. No	1.08 (0.86, 1.36)	0.51
**Best post-transplant response**^**a**^		
CR vs. non-CR	0.50 (0.43, 0.59)	<0.001
**Maintenance therapy**^**a**^		
Yes vs. No	0.69 (0.59, 0.81)	<0.001

*auto-HCT* autologous hematopoietic stem cell transplant, *CI* confidence interval, *CR* complete response, *HCT-CI* hematopoietic cell transplantation-specific comorbidity index, *KRD* carfilzomib, lenalidomide, dexamethasone, *LDH* lactate dehydrogenase, *MRD* minimal residual disease, *R-ISS* Revised International Staging Systems, *ULN* upper limit of normal, *VGPR* very good partial response.

^a^Included in the model as a time-dependent covariate.

**Table 4:** Summary of overall survival – multivariable assessments *(matched patients)*.

Measure	Overall survival	
	Hazard ratio (95% CI)	*p*-value
**CD34**^**+**^ **(×10**^**6**^ **cells/kg)**		
>2.5 vs. ≤2.5	0.42 (0.28, 0.63)^b^	<0.001
**Age at auto-HCT**		
Continuous	1.04 (1.01, 1.06)	0.004
**Year of auto-HCT**		
≥2010 vs. <2010	0.53 (0.30, 0.94)	0.029
**R-ISS**		
II vs. I	1.18 (0.57, 2.45)	0.66
III vs. I	2.18 (0.81, 5.88)	0.12
Unknown vs. I	1.48 (0.73, 3.00)	0.28
**Cytogenetic risk**		
High vs. Standard	1.57 (0.91, 2.72)	0.10
Unknown vs. Standard	2.33 (1.24, 4.37)	0.009
**LDH**		
>ULN vs. Normal	1.92 (1.02, 3.62)	0.042
Unknown vs. Normal	0.75 (0.43, 1.33)	033
**Creatinine**		
>2 vs. ≤2	2.34 (1.35, 4.05)	0.002
Unknown vs. ≤2	4.13 (1.78, 9.58)	<0.001
**Best response**^**a**^		
CR vs. non-CR	0.59 (0.39, 0.90)	0.014
**Maintenance therapy**^**a**^		
Yes vs. No	0.49 (0.31, 0.79)	0.003

*auto-HCT* autologous hematopoietic stem cell transplant, *CI* confidence interval, *CR* complete response, *R-ISS* Revised International Staging Systems, *ULN* upper limit of normal.

^a^Included in the model as a time-dependent covariate.

^b^HR (95% CI) of conditioning regression model; 0.52 (0.31, 0.87); *p* = 0.012.

## Discussion

Despite the widespread use of auto-HCT in patients with MM, the optimal CD34^+^ cell dose to infuse during transplant remains unclear. Current recommendations for CD34^+^ dosing were developed prior to the use of contemporary therapeutic agents and also applied to non-MM patients. In this large cohort of MM patients who received upfront auto-HCT, we showed that a CD34^+^ cell dose of >2.5 × 106 cells/kg is associated with faster hematologic recovery and decreased length of hospitalization, as well as improved PFS and OS, compared to a CD34^+^ cell dose of ≤2.5 × 106 cells/kg. Incremental increase in CD34^+^ dose beyond 2.5 × 10^6^ cells/kg was not associated with any additional benefit. The OS benefit was confirmed by a separate analysis using propensity score matching.

The IMWG consensus statement recommends that a minimum and ideal target of 4 × 10^6^ CD34^+^ cells/kg and 8–10 × 10^6^ CD34^+^ cells/kg, respectively, be collected. This allows most patients to undergo at least two autografts if needed [1]. They advocate for a minimum acceptable dose of 2 × 10^6^ CD34^+^ cells/kg and an optimal dose of 4–6 × 10^6^ CD34^+^ cells/kg for a single transplant. These recommendations were based on older retrospective studies with significant heterogeneity in conditioning regimens and optimal CD34^+^ cell cutoff [2, 19, 20]. They also largely focused on hematologic recovery, rather than survival. Studies have shown that a higher CD34^+^ cell dose was associated with improved PFS and OS after auto-HCT in non-Hodgkin lymphoma and Hodgkin disease [5–8]. However, the role of infused CD34^+^ cell dose on survival in patients with MM is not clear. Moreover, some studies in MM have focused on optimal CD34^+^ cell mobilization rather than optimal CD34^+^ cell dose for infusion, with varying outcomes [21–23].

In a study that included 117 MM patients who underwent auto-HCT, the infused CD34^+^ cell dose showed no impact on neutrophil recovery. However, a significant association was seen between a CD34^+^ cell dose of ≥1.5 × 10^6^ cells/kg and platelet recovery [2]. Conversely, a study with 508 MM patients showed a faster hematologic recovery and reduced hospitalization with CD34^+^ cell dose of ≥6.55 × 10^6^ cells/kg with CD34^+^ cell selection, and ≥ 7.50 × 10^6^ cells/kg without CD34^+^ cell selection. Interestingly, OS, transplant-related mortality, and day-100 response rates were not correlated with CD34^+^ cell dose [3]. A prospective trial at MD Anderson Cancer Center assessed the impact of CD34^+^ cell dose on the outcomes of patients with MM (73%) or light chain amyloidosis who underwent auto-HCT and received either a standard (4–6 × 10^6^ CD34^+^ cells/kg) or high-dose (10–15 × 10^6^ CD34^+^ cells/kg). The trial revealed no significant difference in symptom burden, hematologic recovery, or survival between the two dose groups [4]. This aligns with our results, showing no benefit in survival outcomes beyond an infused dose of 2.5 × 10^6^ CD34^+^ cells/kg. It also highlights that a faster hematologic recovery does not necessarily correlate with better survival. In contrast, a retrospective study from Turkey with 271 patients enrolled between 2003 and 2019, revealed that a CD34^+^ cell dose of ≥5 × 10^6^ CD34^+^ cells/kg was associated with a faster neutrophil and platelet recovery and superior median OS (145 months vs. 103 months; *p* = 0.009) when compared to a dose of <5 × 10^6^ CD34^+^ cells/kg [9]. These contradictory results underscore the need for larger studies in the era of novel therapies. We believe that our current study, which included a large cohort that was treated with contemporary regimens is a step in the right direction.

Previous studies have suggested that the composition and quality of the graft is as important as the dose of CD34^+^ cells [24–26]. In a recent study by our group, the presence of clonal plasma cells (CPC) in the autograft was associated with worse survival outcomes [26]. Furthermore, infusion of CPC-contaminated autografts was associated with inferior PFS in multivariable analysis. Myeloid-derived suppressor cells (MDSC) are also possible contaminants of autologous grafts, and studies have observed a correlation between pre-transplant MDSC and adverse outcomes in MM [27, 28]. Setting lower thresholds for CD34^+^ cell collection and shortening the collection process could potentially reduce the amount of collected and infused detrimental MDSC.

In the present study, patients who received KRD as induction had superior PFS in multivariable analysis compared with recipients of other regimens. Although VRD was the most commonly used induction regimen, several other regimens were also used. Similar to our current study, another single-center study observed improved 5-year PFS (67% vs. 56%, *p* = 0.027) and a trend toward improved OS (90% vs. 80%; *p* = 0.053) in patients who received KRD vs. VRD in newly diagnosed MM patients. This benefit was notable in the high-risk subgroup, which accounted for 49% and 37% of the KRD and VRD groups, respectively [29].

Increasing use of daratumumab in the frontline setting for transplant-eligible MM patients [30–32] has also raised concerns about its impact on stem cell mobilization and collection [33, 34]. Moreover, with greater use of CAR-T cells and ongoing sporadic use of tandem auto-HCT, it is important to define optimal CD34^+^ cell collection and infusion targets in MM. Immune Effector Cell Associated Hematotoxicity (ICAHT) is a common toxicity following anti-BMCA CAR-T cell therapy. In a recent study of 108 MM patients who underwent anti-BMCA CAR-T cell therapy, 60% experienced ICAHT at day 21, 28% of whom received a stem cell boost at a median of 116 days post-CAR-T infusion. Stem cell boost resulted in neutrophil recovery in all patients and improvements in hemoglobin and platelets in the majority [12]. Based on these data, the European Society for Blood and Marrow Transplantation (EBMT) and the European Hematology Association (EHA) jointly recommended best practices for managing ICAHT, suggesting autologous stem cell boosts for ≥grade 3 ICAHT beyond day 14 if readily available. Furthermore, there have been several reports of using allogeneic stem cell boosts to mitigate prolonged cytopenias after CAR-T therapy [35, 36]. These factors underscore a future broader use of CD34^+^ cells and their optimal dose, and some of the collected cells for auto-HCT could be set aside for potential ICAHT in the future.

Our study has inherent limitations of a retrospective analysis, including selection bias, treatment heterogeneity, a relatively small number of patients in the low CD34^+^ dose cohort, and missing data. Notwithstanding these limitations, this is one of the largest studies to evaluate the association between CD34^+^ cell dose and survival outcomes in patients with MM undergoing auto-HCT. Bottom of Form

In summary, our study showed that a CD34^+^ cell dose of >2.5 × 10^6^ CD34^+^ cells/kg, compared to ≤2.5 × 10^6^ CD34^+^ cells/kg, is associated with optimal hematologic engraftment and better survival.

## Supplementary information

Supplementary Table 1

Supplementary Table 2

Supplementary Table 3

Supplementary Table 4

Supplementary Table 5

Supplementary Table 6

## Data Availability

The data that support the findings of this study are available on request from the corresponding author.
