# Supplementary material for: Optimal infused CD34+ cell dose in multiple myeloma patients undergoing upfront autologous hematopoietic stem cell transplantation
Source: Blood Cancer J. 2024 Oct 31;14(1):189. doi: 10.1038/s41408-024-01165-w (PMC11527997; doi:10.1038/s41408-024-01165-w)
Supplement: Supplementary file 1 — Supplementary Table 1 [file 41408_2024_1165_MOESM1_ESM.docx]

**Supplementary Table 1: Patient characteristics – All patients and by CD34^+^ Dose Group, *matched patients***

|  |  | **CD34^+^ Dose Group** | | |
| --- | --- | --- | --- | --- |
| **Measure** | **All**  **(n=285)** | **≤2.5 x 10^6^ cells/kg (N=95)** | **>2.5 x 10^6^ cells/kg (N=190)** | **p-value** |
| **Gender, n (%)** |  |  |  |  |
| Male | 152 (53) | 50 (53) | 102 (54) | 0.90 |
| Female | 133 (47) | 45 (47) | 88 (46) |  |
| **Age at auto-HCT (years)** |  |  |  |  |
| Median (range) | 63.3 (32.2 - 80.6) | 63.2 (32.2 - 77.6) | 63.4 (37.7 - 80.6) | 0.72 |
| **Race, n (%)** |  |  |  |  |
| Black | 48 (17) | 11 (12) | 37 (20) | 0.13 |
| Non-black | 231 (83) | 82 (88) | 149 (80) |  |
| Unknown | 6 | 2 | 4 |  |
| **Year of auto-HCT, n (%)** |  |  |  |  |
| < 2010 | 36 (13) | 11 (12) | 25 (13) | 0.85 |
| ≥ 2010 | 249 (87) | 84 (88) | 165 (87) |  |
| **R-ISS, n (%)** |  |  |  |  |
| I | 51 (29) | 12 (19) | 39 (36) | **0.048** |
| II | 97 (56) | 40 (63) | 57 (52) |  |
| III | 25 (14) | 12 (19) | 13 (12) |  |
| Unknown | 112 | 31 | 81 |  |
| **Light chain type, n (%)** |  |  |  |  |
| Kappa | 181 (64) | 59 (63) | 122 (65) | 0.46 |
| Lambda | 101 (36) | 34 (36) | 67 (35) |  |
| Biclonal | 1 (<1) | 1 (1) | 0 |  |
| Unknown | 2 | 1 | 1 |  |
| **Cytogenetic risk, n (%)** |  |  |  |  |
| Standard | 189 (73) | 59 (69) | 130 (75) | 0.37 |
| High | 69 (27) | 26 (31) | 43 (25) |  |
| Unknown | 27 | 10 | 17 |  |
| **LDH, n (%)** |  |  |  |  |
| Normal | 158 (83) | 49 (82) | 109 (83) | 0.84 |
| > ULN | 33 (17) | 11 (18) | 22 (17) |  |
| Unknown | 94 | 35 | 59 |  |
| **Creatinine, n (%)** |  |  |  |  |
| ≤ 2 | 218 (81) | 70 (78) | 148 (83) | 0.32 |
| > 2 | 50 (19) | 20 (22) | 30 (17) |  |
| Unknown | 17 | 5 | 12 |  |
| **HCT-CI, n (%)** |  |  |  |  |
| ≤ 3 | 208 (73) | 69 (73) | 139 (73) | 1.00 |
| > 3 | 77 (27) | 26 (27) | 51 (27) |  |
| **Chemotherapy-Mobilization, n (%)** |  |  |  |  |
| No | 242 (89) | 83 (90) | 159 (88) | 0.69 |
| Yes | 31 (11) | 9 (10) | 22 (12) |  |
| Unknown | 12 | 3 | 9 |  |
| **Induction treatment, n (%)** |  |  |  |  |
| KRD | 43 (15) | 17 (18) | 26 (14) | 0.38 |
| Imid+Dexa | 24 (8) | 10 (11) | 14 (7) | 0.37 |
| VTD | 15 (5) | 5 (5) | 10 (5) | 1.00 |
| VCD | 37 (13) | 11 (12) | 26 (14) | 0.71 |
| VD | 34 (12) | 10 (11) | 24 (13) | 0.70 |
| VRD | 87 (31) | 27 (28) | 60 (32) | 0.68 |
| **Conditioning regimen, n (%)** |  |  |  |  |
| Mel | 257 (90) | 84 (88) | 173 (91) | 0.53 |
| Bu/Mel based | 28 (10) | 11 (12) | 17 (9) |  |
| **Response prior to auto-HCT, n (%)** |  |  |  |  |
| sCR/CR | 38 (13) | 16 (17) | 22 (12) | 0.27 |
| VGPR | 129 (45) | 40 (42) | 89 (47) | 0.53 |
| PR | 116 (41) | 38 (40) | 78 (41) | 0.90 |
| SD | 2 (1) | 1 (1) | 1 (1) | 1.00 |
| **MRD status prior to auto-HCT, n (%)** |  |  |  |  |
| Negative | 87 (45) | 33 (48) | 54 (43) | 0.55 |
| Positive | 108 (55) | 36 (52) | 72 (57) |  |
| Not done/unknown | 90 | 26 | 64 |  |
| **Maintenance therapy, n (%)** |  |  |  |  |
| No | 66 (23) | 22 (23) | 44 (23) | 1.00 |
| Yes | 219 (77) | 73 (77) | 146 (77) |  |
| Rev +/- Dexa | 159 (73) | 51 (70) | 108 (74) | 0.70 |
| Non-Rev | 46 (21) | 16 (22) | 30 (21) |  |
| Rev/Elo | 14 (6) | 6 (8) | 8 (5) |  |

Abbreviations: auto-HCT = autologous hematopoietic stem cell transplant; Bu/Mel = busulfan, melphalan; CR = complete response; Dexa = dexamethasone; Elo = elotuzumab; IMiD = immunomodulatory drug; KRD = carfilzomib, lenalidomide, dexamethasone; LDH = lactate dehydrogenase; Mel = melphalan; MRD = minimal residual disease; n= number; PR = partial response; R-ISS = Revised International Staging Systems; Rev = lenalidomide, sCR = stringent complete response; SD = stable disease; ULN = upper limit of normal; VCD = bortezomib, cyclophosphamide, dexamethasone; VD = bortezomib, dexamethasone; VGPR = very good partial response; VRD =  bortezomib, lenalidomide, dexamethasone; VTD = bortezomib, thalidomide, dexamethasone.
