# Supplementary material for: Optimal infused CD34+ cell dose in multiple myeloma patients undergoing upfront autologous hematopoietic stem cell transplantation
Source: Blood Cancer J. 2024 Oct 31;14(1):189. doi: 10.1038/s41408-024-01165-w (PMC11527997; doi:10.1038/s41408-024-01165-w)
Supplement: Supplementary file 2 — Supplementary Table 2 [file 41408_2024_1165_MOESM2_ESM.docx]

###### **Supplementary Table 2. Summary of Engraftment Outcomes – All Patients and by CD34^+^ Dose Group**

|  | | **CD34^+^ Dose Group** | | |
| --- | --- | --- | --- | --- |
| **Measure, median (range)** | **All (N=2479)** | **≤2.5 x 10^6^ cells/kg** | **> 2.5 x 10^6^ cells/kg** | **p-value** |
|  |  | **(N=95)** | **(N=2384)** |  |
| **Days to ANC 500 recovery** | 11 (8 - 375) | 12 (10 - 14) | 11 (8 - 375) | **< 0.001** |
| **Days to platelet 20K recovery** | 11 (0 - 110) | 14 (9 - 29) | 11 (0 - 110) | **< 0.001** |
| **Days to platelet 50K recovery** | 14 (0 - 722) | 18 (11 - 95) | 14 (0 - 722) | **< 0.001** |
| **Number of RBC transfusions** | 1 (0 - 25) | 2 (0 - 14) | 1 (0 - 25) | **< 0.001** |
| **Number of platelet transfusion** | 2 (0 - 178) | 3 (1 - 178) | 2 (0 - 118) | **< 0.001** |
| **Days to last RBC transfusion** | 2 (0 - 178) | 3 (1 - 178) | 2 (0 - 118) | **< 0.001** |
| **Days to last platelet transfusion** | 2 (0 - 178) | 3 (1 - 178) | 2 (0 - 118) | **< 0.001** |
