# Supplementary material for: Optimal infused CD34+ cell dose in multiple myeloma patients undergoing upfront autologous hematopoietic stem cell transplantation
Source: Blood Cancer J. 2024 Oct 31;14(1):189. doi: 10.1038/s41408-024-01165-w (PMC11527997; doi:10.1038/s41408-024-01165-w)
Supplement: Supplementary file 3 — Supplementary Table 3 [file 41408_2024_1165_MOESM3_ESM.docx]

**Supplementary Table 3: Summary of Response and Minimal Residual Disease after auto-HCT, *matched patients***

| **Outcome, n (%)** | **CD34^+^ Dose Group** | | | |
| --- | --- | --- | --- | --- |
|  | **All**  **(n=285)** | **≤2.5 x 10^6^ cells/kg (N=95)** | **>2.5 x 10^6^ cells/kg (N=190)** | **p-value** |
| **Day100 response** |  |  |  |  |
| sCR/CR | 92 (32) | 32 (34) | 60 (32) | 0.42 |
| VGPR | 136 (48) | 44 (46) | 92 (49) |  |
| PR | 52 (18) | 16 (17) | 36 (19) |  |
| SD | 1 (<1) | 1 (1) | 0 |  |
| PD | 3 (1) | 2 (2) | 1 (1) |  |
| Early death | 1 | 0 | 1 |  |
| **Best response** |  |  |  |  |
| sCR/CR | 145 (51) | 47 (49) | 98 (52) | 0.67 |
| VGPR | 111 (39) | 38 (40) | 73 (39) |  |
| PR | 25 (9) | 8 (8) | 17 (9) |  |
| PD | 3 (1) | 2 (2) | 1 (1) |  |
| Early death | 1 | 0 | 1 |  |
| **MRD negative ≥VGPR at best post-transplant response** |  |  |  |  |
| Yes | 54 (71) | 21 (72) | 33 (70) | 1.00 |
| No | 22 (29) | 8 (28) | 14 (30) |  |
| MRD not performed | 209 | 66 | 143 |  |

Abbreviations: auto-HCT = autologous hematopoietic stem cell transplant; CR = complete response; MRD =

minimal residual disease; n= number; PD = progressive disease; PR= partial response; sCR = stringent

complete response; SD = stable disease; VGPR = very good partial response.
