# Supplementary material for: Optimal infused CD34+ cell dose in multiple myeloma patients undergoing upfront autologous hematopoietic stem cell transplantation
Source: Blood Cancer J. 2024 Oct 31;14(1):189. doi: 10.1038/s41408-024-01165-w (PMC11527997; doi:10.1038/s41408-024-01165-w)
Supplement: Supplementary file 4 — Supplementary Table 4 [file 41408_2024_1165_MOESM4_ESM.docx]

**Supplementary Table 4: Summary of Progression-Free and Overall Survival – Univariate Assessments**

|  | **Progression-Free Survival** | | |
| --- | --- | --- | --- |
| **CD34^+^ (x 10^6^ cells/kg)** | **Median (95% CI) in months** | **Hazard Ratio (95% CI)** | **p-value^a^** |
| ≤ 2.5 | 31.6 (25.0, 45.0) | ref |  |
| > 2.5 | 43.6 (40.8, 47.4) | 0.72 (0.56, 0.93) | **0.011** |
| ≤ 3 | 48.2 (37.4, 59.9) | ref |  |
| > 3 | 43.0 (39.7, 45.2) | 1.03 (0.87, 1.21) | 0.75 |
| ≤ 4 | 46.0 (41.4, 52.8) | ref |  |
| > 4 | 40.8 (38.3, 44.6) | 1.07 (0.96, 1.19) | 0.21^b^ |
| ≤ 5 | 43.5 (40.2, 46.4) | ref |  |
| > 5 | 43.1 (37.3, 50.0) | 0.98 (0.87, 1.11) | 0.79 |
| ≤ 6 | 43.5 (40.2, 46.2) | ref |  |
| > 6 | 43.1 (35.1, 52.6) | 1.02 (0.88, 1.20) | 0.76 |
|  | **Overall Survival** | | |
| **CD34^+^ (x 10^6^ cells/kg)** | **Median (95% CI) (in months)** | **Hazard Ratio (95% CI)** | **p-value** |
| ≤ 2.5 | 76.4 (58.0, 79.5) | ref |  |
| > 2.5 | 108.2 (102.6, 114.4) | 0.53 (0.40, 0.72) | **< 0.001** |
| ≤ 3 | 85.7 (76.6, 102.3) | ref |  |
| > 3 | 108.4 (103.3, 115.0) | 0.83 (0.67, 1.02) | 0.08 |
| ≤ 4 | 100.7 (91.3, 109.1) | ref |  |
| > 4 | 111.3 (103.8, 122.7) | 0.89 (0.78, 1.02) | 0.10 |
| ≤ 5 | 103.8 (95.9, 110.2) | ref |  |
| > 5 | 113.6 (102.6, 138.1) | 0.89 (0.76, 1.03) | 0.13^c^ |
| ≤ 6 | 104.6 (97.9, 110.8) | ref |  |
| > 6 | 119.0 (101.9, 145.8) | 0.90 (0.73, 1.10) | 0.30 |

^a^ P-values are both from the log-rank test and Wald chi-squared test.

^b^ Log-rank p-value = 0.20.

^c^ Log-rank p-value = 0.12.

Abbreviations: CI = confidence interval; *ref* = reference group.
