# Supplementary material for: Optimal infused CD34+ cell dose in multiple myeloma patients undergoing upfront autologous hematopoietic stem cell transplantation
Source: Blood Cancer J. 2024 Oct 31;14(1):189. doi: 10.1038/s41408-024-01165-w (PMC11527997; doi:10.1038/s41408-024-01165-w)
Supplement: Supplementary file 5 — Supplementary Table 5 [file 41408_2024_1165_MOESM5_ESM.docx]

**Supplementary Table 5: Summary of Progression-Free Survival – Univariate Assessments**

| **Measure** | **Hazard Ratio**  **(95% CI)** | **p-value** |
| --- | --- | --- |
| **CD34^+^ (x 10^6^ cells/kg)** |  |  |
| > 2.5 vs. ≤ 2.5 | 0.72 (0.56, 0.93) | **0.011** |
| Continuous | 1.00 (0.97, 1.03) | 0.94 |
| **Gender** |  |  |
| Female vs. Male | 0.96 (0.87, 1.07) | 0.48 |
| **Age at auto-HCT** |  |  |
| Continuous | 1.01 (1.00, 1.02) | **< 0.001** |
| **Race** |  |  |
| Non-black vs. Black | 0.95 (0.83, 1.09) | 0.44 |
| **Year of auto-HCT** |  |  |
| ≥ 2010 vs. < 2010 | 0.68 (0.61, 0.76) | **< 0.001** |
| **R-ISS** |  |  |
| II vs. I | 1.38 (1.19, 1.60) | **< 0.001** |
| III vs. I | 2.30 (1.82, 2.92) | **< 0.001** |
| **Light chain type** |  |  |
| Lambda vs. Kappa | 1.27 (1.14, 1.41) | **< 0.001** |
| Biclonal vs. Kappa | 0.77 (0.36, 1.61) | 0.49 |
| **Cytogenetic risk** |  |  |
| High vs. Standard | 1.65 (1.45, 1.87) | **< 0.001** |
| **LDH** |  |  |
| > ULN vs. Normal | 1.28 (1.07, 1.53) | **0.006** |
| **Creatinine** |  |  |
| > 2 vs. ≤ 2 | 1.05 (0.90, 1.22) | 0.55 |
| **HCT-CI** |  |  |
| > 3 vs. ≤ 3 | 1.08 (0.95, 1.22) | 0.25 |
| **Chemotherapy- mobilization** |  |  |
| Yes vs. No | 1.28 (1.11, 1.49) | **0.001** |
| **Induction treatment** |  |  |
| Imid+Dexa vs. KRD | 2.05 (1.63, 2.59) | **< 0.001** |
| VTD vs. KRD | 1.81 (1.34, 2.43) | **< 0.001** |
| VCD vs. KRD | 1.60 (1.25, 2.04) | **< 0.001** |
| VD vs. KRD | 1.67 (1.32, 2.12) | **< 0.001** |
| VRD vs. KRD | 1.39 (1.11, 1.74) | **0.004** |
| Other vs. KRD | 1.67 (1.30, 2.14) | **< 0.001** |
| **Conditioning regimen** |  |  |
| Bu/Mel based vs. Mel | 0.93 (0.78, 1.10) | 0.38 |
| Other vs. Mel | 1.17 (0.93, 1.47) | 0.17 |
| **Response prior to auto-HCT** |  |  |
| VGPR vs. sCR/CR | 1.43 (1.18, 1.75) | **< 0.001** |
| PR vs. sCR/CR | 1.68 (1.38, 2.04) | **< 0.001** |
| SD vs. sCR/CR | 1.58 (1.11, 2.25) | **0.012** |
| **MRD status prior to auto-HCT** |  |  |
| Positive vs Negative | 1.55 (1.32, 1.81) | **< 0.001** |
| **MRD negative ≥VGPR prior to auto-HCT** |  |  |
| No vs. Yes | 1.86 (1.59, 2.17) | **< 0.001** |
| **Best post-transplant response^a^** |  |  |
| CR vs. non-CR | 0.36 (0.32, 0.40) | **< 0.001** |
| **MRD status at best post-transplant response^a^** |  |  |
| Negative vs. Positive | 0.54 (0.42, 0.70) | **< 0.001** |
| **Maintenance therapy^a^** |  |  |
| Yes vs. No | 0.52 (0.47, 0.58) | **< 0.001** |
| Rev+/-Dexa vs non-Rev | 0.75 (0.64, 0.89) | **< 0.001** |

Abbreviations: auto-HCT = autologous hematopoietic stem cell transplant; Bu/Mel = busulfan, melphalan; CI = confidence interval; CR = complete response; Dexa = dexamethasone; HCT-CI = hematopoietic cell transplantation-specific comorbidity index; IMiD = immunomodulatory drug; KRD = carfilzomib, lenalidomide, dexamethasone; LDH = lactate dehydrogenase; Mel = melphalan; MRD = minimal residual disease; PR = partial response; R-ISS = Revised International Staging Systems; Rev = lenalidomide; sCR = stringent complete response; SD = stable disease; ULN = upper limit of normal; VCD = bortezomib, cyclophosphamide, dexamethasone; VD = bortezomib, dexamethasone; VGPR = very good partial response; VRD =  bortezomib, lenalidomide, dexamethasone; VTD = bortezomib, thalidomide, dexamethasone.

^a^Included in the model as a time-dependent covariate.
