# Supplementary material for: Optimal infused CD34+ cell dose in multiple myeloma patients undergoing upfront autologous hematopoietic stem cell transplantation
Source: Blood Cancer J. 2024 Oct 31;14(1):189. doi: 10.1038/s41408-024-01165-w (PMC11527997; doi:10.1038/s41408-024-01165-w)
Supplement: Supplementary file 6 — Supplementary Table 6 [file 41408_2024_1165_MOESM6_ESM.docx]

**Supplementary Table 6: Summary of Overall Survival – Univariate Assessments**

| **Measure** | **Hazard Ratio**  **(95% CI)** | **p-value** |
| --- | --- | --- |
| **CD34^+^ (x 10^6^ cells/kg)** |  |  |
| > 2.5 vs. ≤ 2.5 | 0.53 (0.40, 0.72) | **< 0.001** |
| Continuous | 0.96 (0.93, 1.00) | 0.06 |
| **Gender** |  |  |
| Female vs. Male | 0.92 (0.80, 1.05) | 0.22 |
| **Age at auto-HCT** |  |  |
| Continuous | 1.03 (1.02, 1.03) | **< 0.001** |
| **Race** |  |  |
| Non-black vs. Black | 1.16 (0.97, 1.39) | 0.11 |
| **Year of auto-HCT** |  |  |
| ≥ 2010 vs. < 2010 | 0.85 (0.73, 0.99) | **0.038** |
| **R-ISS** |  |  |
| II vs. I | 1.72 (1.37, 2.14) | **< 0.001** |
| III vs. I | 4.11 (3.03, 5.57) | **< 0.001** |
| **Light chain type** |  |  |
| Lambda vs. Kappa | 1.30 (1.13, 1.50) | **< 0.001** |
| Biclonal vs. Kappa | 1.00 (0.41, 2.41) | 1.00 |
| **Cytogenetic risk** |  |  |
| High vs. Standard | 2.06 (1.74, 2.44) | **< 0.001** |
| **LDH** |  |  |
| > ULN vs. Normal | 1.56 (1.24, 1.95) | **< 0.001** |
| **Creatinine** |  |  |
| > 2 vs. ≤ 2 | 1.37 (1.14, 1.65) | **< 0.001** |
| **HCT-CI** |  |  |
| > 3 ≤ 3 | 1.44 (1.24, 1.69) | **< 0.001** |
| **Chemotherapy- mobilization** |  |  |
| Yes vs. No | 1.05 (0.86, 1.28) | 0.64 |
| **Induction treatment** |  |  |
| ImiD+Dexa vs. KRD | 1.43 (1.00, 2.06) | 0.052 |
| VTD vs. KRD | 1.55 (1.03, 2.35) | **0.037** |
| VCD vs. KRD | 1.55 (1.06, 2.26) | **0.022** |
| VD vs. KRD | 1.47 (1.02, 2.13) | **0.038** |
| VRD vs. KRD | 1.17 (0.82, 1.68) | 0.38 |
| Other vs. KRD | 1.54 (1.06, 2.25) | **0.025** |
| **Conditioning regimen** |  |  |
| Bu/Mel based vs. Mel | 1.03 (0.82, 1.29) | 0.82 |
| Other vs. Mel | 1.29 (0.98, 1.69) | 0.07 |
| **Response prior to auto-HCT** |  |  |
| VGPR vs. sCR/CR | 1.40 (1.08, 1.82) | **0.011** |
| PR vs. sCR/CR | 1.40 (1.08, 1.81) | **0.012** |
| SD vs. sCR/CR | 1.35 (0.85, 2.15) | 0.21 |
| **MRD status prior to auto-HCT** |  |  |
| Positive vs. Negative | 1.02 (0.81, 1.28) | 0.87 |
| **MRD negative ≥VGPR prior to auto-HCT** |  |  |
| No vs. Yes | 1.32 (1.07, 1.62) | **0.009** |
| **Best response^a^** |  |  |
| CR vs. non-CR | 0.42 (0.37, 0.49) | **< 0.001** |
| **MRD status at best post-transplant response^a^** |  |  |
| Negative vs. Positive | 0.83 (0.55, 1.25) | 0.37 |
| **Maintenance therapy^a^** |  |  |
| Yes vs. No | 0.60 (0.53, 0.69) | **< 0.001** |
| Rev+/-Dexa vs non-Rev | 0.81 (0.65, 1.01) | 0.06 |

Abbreviations: auto-HCT = autologous hematopoietic stem cell transplant; Bu/Mel = busulfan, melphalan; CR = complete response; Dexa = dexamethasone; HCT-CI = hematopoietic cell transplantation-specific comorbidity index; ImiD = immunomodulatory drug; KRD = carfilzomib, lenalidomide, dexamethasone; LDH = lactate dehydrogenase; Mel = melphalan; MRD = minimal residual disease; PR = partial response; R-ISS = Revised International Staging Systems; Rev = lenalidomide; sCR = stringent complete response; SD = stable disease; ULN = upper limit of normal; VCD = bortezomib, cyclophosphamide, dexamethasone; VD = bortezomib, dexamethasone; VGPR = very good partial response; VRD =  bortezomib, lenalidomide, dexamethasone; VTD = bortezomib, thalidomide, dexamethasone.

^a^Included in the model as a time-dependent covariate.
